# Dehydrozingerone protects temozolomide-induced cognitive impairment in normal and C6 glioma rats besides enhancing its anticancer potential

**DOI:** 10.1007/s13205-020-02427-7

**Published:** 2020-09-17

**Authors:** Nandini Pathak, Sri Pragnya Cheruku, Vanishree Rao, R. J. A. Vibhavari, Suhani Sumalatha, Karthik Gourishetti, C. Mallikarjuna Rao, Nitesh Kumar

**Affiliations:** 1 Department of Pharmacology, Manipal College of Pharmaceutical Sciences, Manipal Academy of Higher Education, Manipal, Karnataka 576104 India; 2 Department of Anatomy, Kasturba Medical College, Manipal Academy of Higher Education, Manipal, Karnataka 576104 India

**Keywords:** C6 glioblastoma, Dehydrozingerone, Cognition, Temozolomide

## Abstract

Considering the cognitive impairment induced by temozolomide (TMZ) in glioblastoma survivors, the present study was aimed to evaluate the protective effect of dehydrozingerone (DHZ) against TMZ-induced cognitive impairment (chemobrain) and C6 cell line-induced glioma in male Wistar rats. In both chemobrain and glioma models, TMZ was administered at a dose of 18 mg/kg i.v every 5th day and DHZ at a dose of 100 mg/kg p.o. daily. Additionally, glioma was induced by intracerebral injection of 5 × 10^4^ C6 rat glioma cells in the cortex in the glioma model. Upon disease induction and treatment with TMZ + DHZ, spatial memory was assessed by the Morris water maze (MWM) test and episodic memory by the novel object recognition test (NORT). The induction of glioma was confirmed by histology of the cortex. Hippocampus and frontal cortex were subjected to antioxidant evaluation. Significant loss of spatial and episodic memory was observed with TMZ treatment which was significantly restored by DHZ. DHZ showed significant improvement in oxidative stress markers reversed the histopathological features in the cortex. TMZ-induced elevation of the glutathione level was also reversed by DHZ, indicating the role of DHZ in the reversal of TMZ resistance. In the glioma model, the improvement in cognition by DHZ correlated with the decrease in tumor volume. Altogether, the study results reveal the role of TMZ in worsening the memory and DHZ in reversing it, besides, improving its anticancer potential.

## Introduction

Chemotherapy is one of the oldest treatment modalities aimed at reducing the tumor burden and improving progression-free overall survival after surgery or radiation therapy. Unfortunately, it is ill-famed to cause cognitive deficits in 70% of cancer patients, of which about 50% report a significant decline in cognitive processes. This is mainly due to its non-selective actions affecting both cancer cells and normal cells. Chemotherapy mediated neurotoxicity has gained much importance in recent years, mainly manifested by impaired attention, executive functions, memory, etc., even after cessation of therapy, consequently compromising the quality of life. This is termed as ‘chemo-fog’ or ‘chemo-brain’ (Lucassen 2012).

Glioma/glioblastoma is the most common and aggressive primary tumor of the glial cells manifested by memory problems, headaches, personality changes, seizures, speech problems, and weakness/numbness in the arms, legs, and face. It is the 3rd most cause of death by cancer in patients of 15–34 years of age. Malignant gliomas are the reason for 2.5% of deaths due to cancers. The standard care of treatment includes surgery/radiation (Hanif et al. 2017). Based on large number of clinical trials, the DNA alkylating agent, temozolomide (TMZ) was approved for the management of glioma as adjuvant therapy (Mrugala and Chamberlain 2008). TMZ, an imidazole derivative, is a prodrug that readily converts into an active metabolite, 5-(3-methyl)-1-triazen-1-yl-imidazole-4-carboxamide (MTIC) systemically. This metabolite acts by hypermethylation at GGG DNA sequences and inactivation of repair enzyme O6 methylguanine-DNA methyltransferase (MGMT) which initiates apoptosis, ultimately resulting in glioma cell death (Dietrich et al. 2015b; Barciszewska et al. 2015). TMZ is readily permeable through the blood–brain barrier, as a result, it produces direct toxicity to the normal cells of the brain leading to impaired hippocampal neurogenesis and disruption in the associative learning (Bird et al. 2016). In the adult brain, the hippocampus is responsible for the formation and retention of episodic and spatial memory (Qu et al. 2017; Ganeshpurkar and Saluja 2017; Ramalingayya et al. 2017). Hippocampal neurogenesis in the adult brain occurs by the process of generating new granule cell neurons. This process regulates the maintenance of brain plasticity, learning, and memory. TMZ treatment for glioblastoma is implicated in the impairment of hippocampal neurogenesis showing disruption in theta activity of the brain which determines the decline in learning and memory and disturbance in communication between brain centers, i.e., inter-regional communication, confirming impairment in cognitive ability (Dietrich et al. 2015b). However, the lack of animal models in this context hinders the research on the evaluation of neuroprotective potentials of various protective agents.

Dehydrozingerone (DHZ) is an unsaturated derivative of ginger rhizome and is a by-product of curcumin (Rajakumar and Rao 1993). Curcumin, a component extracted from the rhizome of the plant *Curcuma longa,* has proven antidepressant, antibacterial, anticancer, anti-inflammatory, and antioxidative properties. DHZ is the half analog of curcumin. Both the compounds are styryl ketones with the phenolic group at the para position and the methoxy group at the ortho position to the phenolic group. The major difference being the presence of a 1,3-diketone system in curcumin which is not seen in DHZ. Like curcumin, DHZ has shown anti-cancer, anti-oxidant, anti-inflammatory, cardioprotective, and chemoprotective effects (Ozeki et al. 2012). Thus, the present study was designed to evaluate the protective effect of DHZ in TMZ-induced cognitive impairment in normal (chemobrain) and C6-induced glioblastoma rats.

## Materials and methods

### Materials

TMZ, adrenaline bitartrate, 5,5-dithio-bis-(2-nitrobenzoic acid) (DTNB), and Vanillin were purchased from Sigma-Aldrich, St. Louis, MO, USA. Trypsin and Triton-X 100 were purchased from Himedia lab Pvt. Ltd. (Mumbai, India). Carboxymethyl cellulose (CMC) was purchased from Rankem, India. Sodium Chloride and trichloroacetic acid were purchased from SD Fine Chemicals, India. Disodium EDTA, hydrogen peroxide, disodium hydrogen phosphate dehydrate sodium bicarbonate, potassium dihydrogen phosphate, and disodium hydrogen phosphate were purchased from Himedia Laboratories Ltd, India. Fetal bovine serum and Dulbecco’s modified Eagle’s medium were purchased from Gibco, USA.

### Cell line

C6 glioma cells were procured from National Centre for Cell Science, Pune India. Cells were grown in Dulbecco’s modified Eagle’s medium (DMEM) containing 10% fetal bovine serum. Cells were subcultured and grown to confluence using T24 tissue culture treated flasks. Cultured cells were maintained at 37 °C using a CO_2_ incubator. The number of cells was adjusted to 50,000 cells per 10 µl.

### Animals

12 Weeks old, healthy Male Wistar rats weighing 150–200 g were used in the study to induce cognitive impairment by TMZ in normal (chemobrain) and glioblastoma models. The experiment protocol was approved by the Institutional Animal Ethics Committee (IAEC) (approval no. IAEC/KMC/99/2017) and the experiments were performed according to the guidelines of the CPCSEA, India. The animals were maintained under controlled conditions such as, temperature (23 ± 2 °C) and humidity (50 ± 5%) with free access to food and water, 12/12 h light and dark cycle.

### C6 glioma model

Adult male Wistar rats weighing 200–250 g were fasted for 3 h before the experiment. For the administration of C6 glioma cells, the animal was anesthetized and the head was shaved. The rat head was fixed in the stereotaxic frame through the mouth and ear holder (Jacobs et al. 2011; Kubra et al. 2014). Skin from the midline of the head was cut using surgical scissors and the area was cleaned with a cotton swab. C6 cells were injected into the cerebrum at coordinates (AP: 0.36 mm, ML: 3.6 mm, and DV: 5 mm). A small hole of 1 mm was drilled in the skull according to the mentioned coordinates. Using a Hamilton syringe, 50,000 C6 glioma cells in 10 µl were injected intracerebrally into the brain. The hole of the skull was sealed with sterile dental cement. The skin incision was sutured and betadine was applied on the wound after the suture (Ozeki et al. 2012).

### Synthesis of DHZ

Vanillin 5 g (0.03 mol) was used for the synthesis of DHZ. 5 g of vanillin was dissolved in 40 ml of acetone, 50 ml of Sodium hydroxide (0.5 N) solution was added with continuous stirring, and stirring was continued for 1 h on a magnetic stirrer. The solution was kept at room temperature for the evaporation of excess acetone. 2 N hydrochloric acid was added to the solution with continuous stirring until the yellow precipitate was formed. The crude precipitate was allowed to dry for 24 h. Recrystallization was done with ethanol followed by hot filtration (Bhosale et al. 2011).

### Characterization of DHZ by liquid chromatography–mass spectrometry

The analyte sample was prepared (~ 1 ppm) in methanol: water (70:30) and identification of the analyte was performed using MS (model LTQ-XL, Thermo Scientific) with interchangeable APCI source in positive ionization mode coupled with HPLC (Model: Dionex Ultimate 3000, Thermo Scientific). The mobile phase consisted of methanol: water (70:30) with total run time of 3 min in isocratic flow. The MS condition included APCI vaporizer temperature 400 °C, capillary temperature: 275 °C, sheath gas flow: 50, Aux gas flow: 10, sweep gas flow: 3, source voltage: 6 kV, Source current: 5 µA, capillary voltage: 9 V, Tube lens: 125 V.

### Experimental design

**Fig.1:**
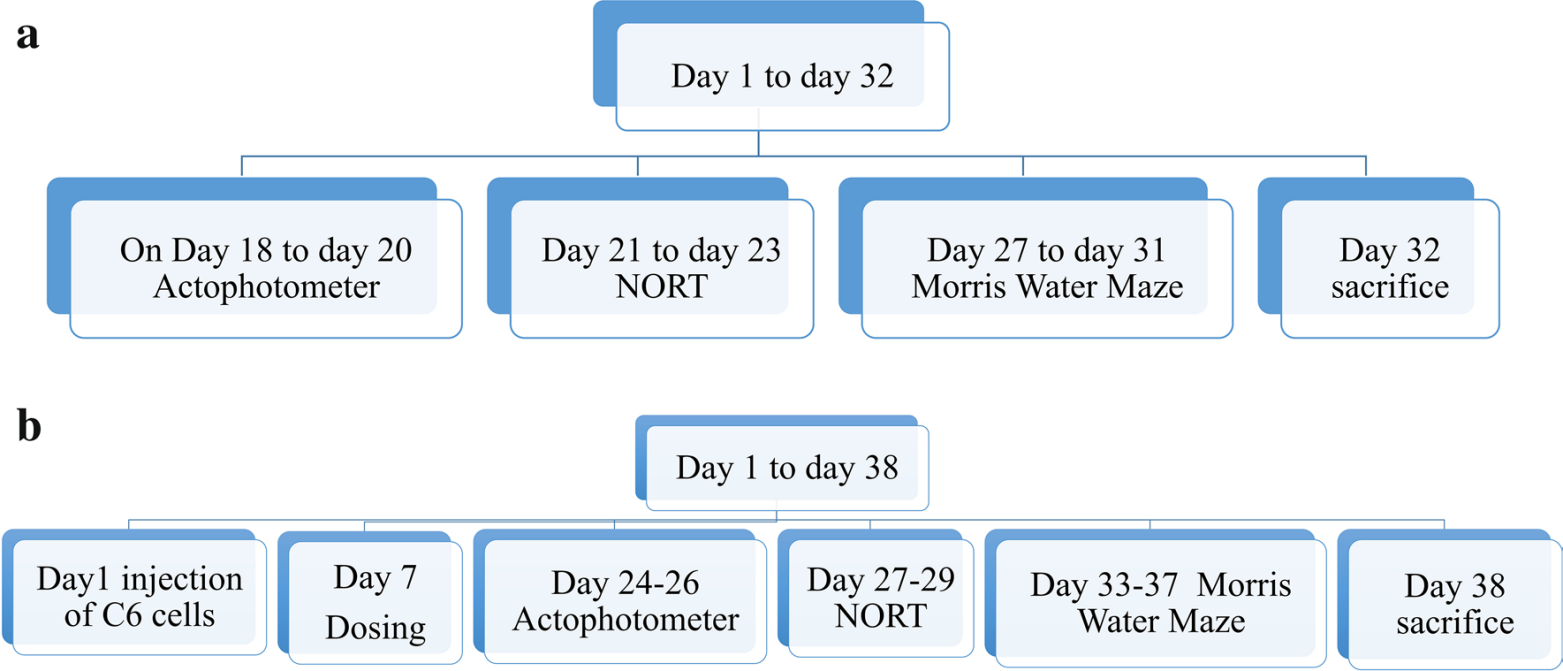
Schematic representation of experimental protocol for temozolomide-induced cognitive impairment (chemobrain) model and Glioblastoma model. Dose of temozolomide: 18 mg/kg i.v. made freshly on the day of administration; dosing schedule: once in 5 days; chemobrain model-day 1, 6, 11, 16, 21, 26 and 31; glioma model: day 7, 12, 17, 22, 29 and 34

All the drug solutions were freshly prepared on the day of administration. Animals were broadly divided into 2 groups namely, temozolomide induced-chemobrain or non-glioma cognitive impairment model and glioma model, to test the effect of temozolomide on cognition in the presence and absence of glioma. Animals in the chemobrain or non-glioma model were subdivided into 3 groups namely, normal control (NC), temozolomide group (TMZ), and temozolomide + dehydrozingerone group (TMZ + DHZ) with 6 animals in each group (Fig. 1a). Glioma model was also subdivided into 4 animal groups (*N* = 6) namely, normal control (NC), glioma or C6 control (C6), C6 + temozolomide group (C6 + TMZ), and C6 + temozolomide + dehydrozingerone group (C6 + TMZ + DHZ) (Fig. 1b). TMZ 18 mg/kg was freshly made on the day of administration and administered intravenously once in 5 days (chemobrain model- day 1, 6, 11, 16, 21, 26 and 31; glioma model: day 7, 12, 17, 22, 29 and 34). DHZ 100 mg/kg dose was prepared in 0.25% w/v CMC and administered orally. Treatment was carried out between 9 a.m. to 4 p.m.

### Actophotometer test

Actophotometer was used for monitoring the locomotor activity of the animals. Each animal was placed in actophotometer for 5 min. The locomotor activity of the animal was recorded with a digital counter. After 30 min of dosing with the respective drug, the animal was placed in the actophotometer for recording the locomotor activity (Saimithra et al. 2018).

### Novel object recognition task (NORT)

NORT was carried out using square boxes of 48 cm (length × breadth × height). The inner portion of the boxes was covered with a black laminate and the boxes were made with plywood. The behavior of animals was monitored with the help of a camera (model QuickC am Pro 9000, Logitech International S.A., Lausanne., Switzerland). Handheld stopwatch and alarm timer were used for behavioral observation (Barnhart et al. 2015).

Three days were given for habituation, familiarization trial, and choice trial with an intertrial interval (ITI) of 12 h (duration of trials 3 min each). After habituation to the test arena, animals were exposed to two similar objects for familiarization. Following this, the rats were subjected to a choice trial with one of the familiar objects replaced by a novel object. The independent expert observers, who were blind to the treatment schedule, used two stop-watches for recording the time spent for the exploration of the two objects. The indices of recognition and discrimination were calculated using formula. The recognition index (RI) is represented by the ratio of exploration time of the novel object (*B*) to the total exploration time of the animal to both novel and familiar objects (*A*_3_ + *B*) i.e., RI = *B*/(*A*_3_ + *B*). The discrimination index (DI) was calculated by the difference between the exploration time of the novel object (*B*) and familiar object (*A*_3_) in the test phase i.e., DI = *B* – *A*_3_ (Cheruku et al. 2018).

### Morris water maze test

Morris Water Maze contains a circular pool of 150 cm diameter, filled with water. The temperature of the water was kept below the body temperature of the animal. The pool was divided into four quadrants. Inside the pool, the hidden platform was kept for the animals to escape. The pool water was made opaque with milk powder to hide the platform. The movement of each animal was captured using a video camera and data were analyzed by software. Animals were trained in four sessions (acquisition trial) to identify the hidden platform in the target quadrant. In the retention trail, the platform was removed and animals’ movements were recorded for 60 s. Parameters total zone entries, average speed, path efficiency, and escape latency to reach the platform were calculated using the software (Nayak et al. 2017; Nampoothiri et al. 2017).

### Oxidative stress markers and AchE activity estimation

The oxidative stress markers such as catalase (Aebi 1984), superoxide dismutase (SOD) (Misra and Fridovich 1972), lipid peroxidation (LPO) by malondialdehyde estimation (MDA) (Rai et al. 2019), GSH (Rai et al. 2019), total protein levels and AchE activity (Ellman et al. 1961) were estimated in the hippocampus and frontal cortex region of the brain, as per our routine laboratory UV spectrometric and colorimetric methods.

### Statistical analysis

Graph pad prism version 6.01 was used for the statistical analysis. All data are expressed as mean and SEM of six samples. Data were analyzed by One-Way ANOVA using the Tukey’s post hoc test.

## Results

### Synthesis and characterization of DHZ

**Fig. 2:**
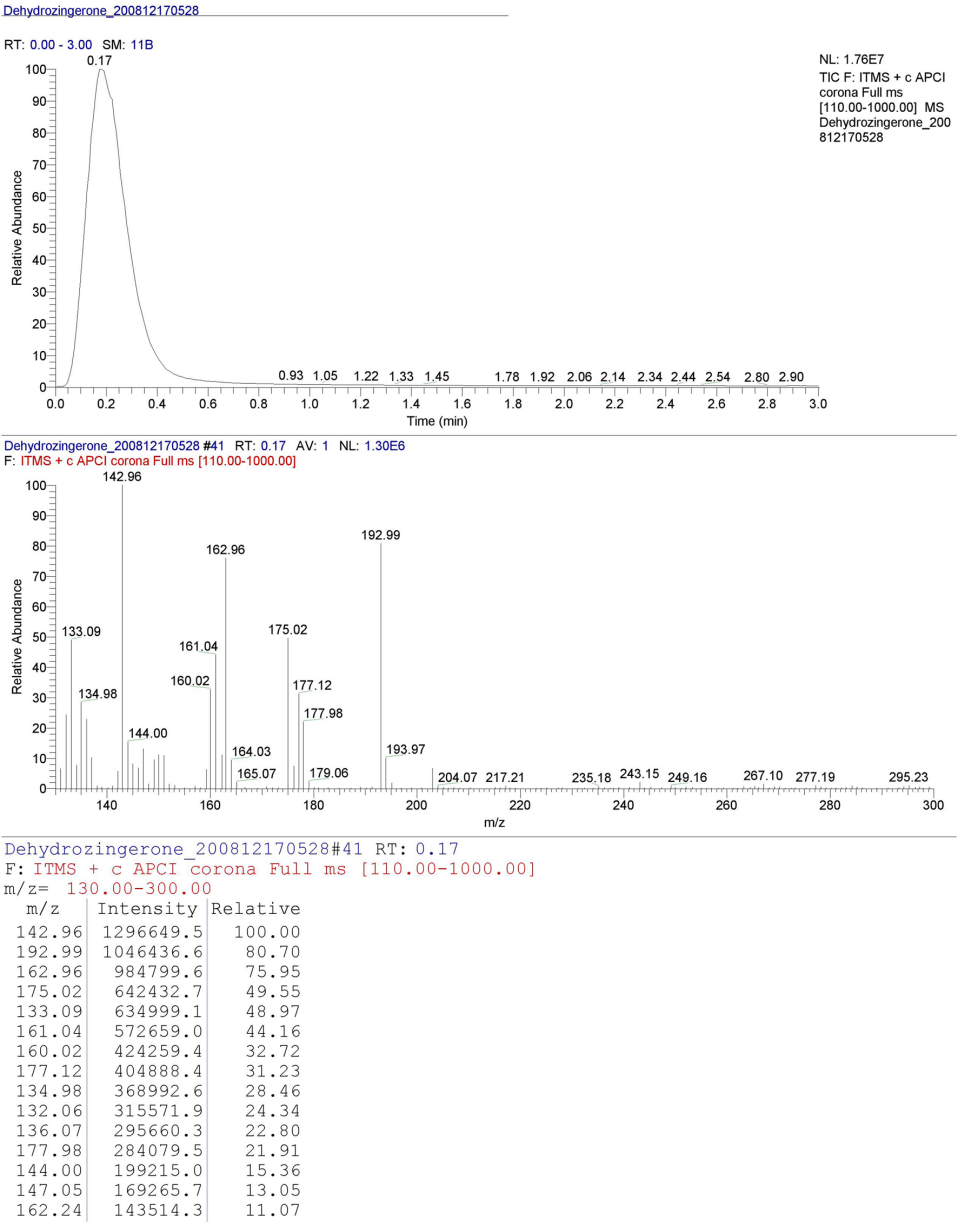
LC–MS spectrum of dehydrozingerone. LCMS-analysis showed the molecular weight of dehydrozingerone (DHZ) as parent ion peak [M + 1]− 192.99 *m/z* with 80% relative intensity. The base peak fragments were found at *m/z* 172 and 142 which confirmed the synthesized product to be DHZ

Synthesis of DHZ was done using vanillin as a starting material. The process resulted in a yellowish-orange colored product that was analyzed by LC–MS, for characterization and evaluation of purity. The parent ion peak was obtained as [M + 1] 192.99 *m/z* with 80% relative intensity consistent with the known molecular weight of DHZ, and the base peak fragments at *m/z* 172 and 142 confirmed the product to be DHZ (Fig. 2).

### Temozolomide-induced cognitive impairment in chemobrain model

#### Locomotor activity

The locomotor activity of the normal animal was assessed by the actophotometer test. No significant change in locomotor activity was observed within various groups (Table 1).

**Table 1:** Effect of treatments on locomotor activity in rats of chemobrain and glioma models

Treatment	Chemobrain model	Glioma model
	(mean ± SEM)	(mean ± SEM)
NC	382.8 ± 19.84	365 ± 12.36
C6 control	–	139 ± 12.63^aaa^
TMZ	331.9 ± 24.61	297.8 ± 29.66^bb^
DHZ + TMZ	406.9 ± 19.26	417.3 ± 26.31^bb,c^

In the glioma model, C6 was injected into all the groups except normal control rats. Locomotor activity was monitored using an actophotometer with a digital counter for 5 min. No significant change was observed in locomotor activity observed among the groups in the chemobrain model. However, a significant change was observed in the glioma model and indicated in the table as ^aaa^*P* < 0.001 compared to normal control; ^bb^*P* < 0.01 compared to C6 control; ^c^*P* < 0.05 compared to TMZ control. The symbols used in the tables are NC: normal control; C6: C6 Glioma control; TMZ: temozolomide; DHZ + TMZ: dehydrozingerone + temozolomide

#### Novel object recognition test

The recognition index is the ratio of the exploration of the novel object to the total exploration time of the animal to both the objects. TMZ group animals showed a significant (0.27 ± 0.08) decline in exploration time with a novel object, when compared to normal control animals (0.62 ± 0.07). The treatment group, DHZ + TMZ showed a significant (0.69 ± 0.09) increase in the exploration time as compared to the TMZ group (Table 2).

**Table 2:** Effect of treatments on NORT parameters in chemobrain and glioma model

Groups	Chemobrain model		Glioma model	
	Recognition index	Discriminative index	Recognition index	Discriminative index
NC	0.62 ± 0.07	3.55 ± 0.5	0.62 ± 0.067	3.55 ± 0.57
C6 control	–	–	0.09 ± 0.09^aa^	− 0.51 ± 0.3^a^
TMZ	0.27 ± 0.08^a^	− 3.07 ± 0.65^a^	0.26 ± 0.10	− 0.69 ± 0.29
DHZ + TMZ	0.69 ± 0.09^bb^	4.11 ± 0.68^b^	0.48 ± 0.07^c^	3.23 ± 0.60^b,c^

NC: NORT assay was conducted with an inter-trial interval of 12 h. In glioma rats model C6 was injected to all the groups except Normal control. The grouping symbols are NC: normal control; C6: C6 Glioma control; TMZ: temozolomide; DHZ + TMZ: dehydrozingerone + temozolomide. All the values are expressed as mean ± SEM of six samples. The differences in the recognition and discriminative indices are represented by a symbol, where ^a^*P* < 0.05 compared to normal control; ^aa^*P* < 0.01 compared to normal control; ^b^*P* < 0.05 compared to TMZ control; ^bb^*P* < 0.01 compared to TMZ control; ^c^*P* < 0.05 compared to C6 control

The discrimination index is the difference in the exploration time of the novel object and the familiar object. TMZ group animals had shown a significant decrease (− 3.066 ± 0.65) in the discrimination of novel objects aginst the familiar object in comparison to normal control animals (3.55 ± 0.5). The treatment group, DHZ + TMZ (4.11 ± 0.68) had shown a significant increase in the discrimination of novel objects from the familiar object as compared to the TMZ control group (Table 2).

#### Morris water maze test

Significant decrease in total zones entries (29.33 ± 3.76) and path efficiency (0.013 ± 0.002) were observed in the TMZ group in comparison with the normal control group (53.0 ± 3.46 and 0.027 ± 0.003), whereas TMZ + DHZ treatment had shown significant improvement in total zonal entries (48.67 ± 4.33) with a slight increase in path efficiency (0.015 ± 0.002) when compared to TMZ group (Table 3).

**Table 3:** Effect on Morris water maze test parameters in chemobrain and glioma model

Groups	Total zones entries (*n*)	Average speed (m/s)	Path efficiency	Escape latency (S)
Chemobrain model				
NC	53.0 ± 3.46	1.74 ± 0.06	0.027 ± 0.003	7.05 ± 0.83
TMZ	29.33 ± 3.76^a^	1.20 ± 0.04^a^	0.013 ± 0.002^a^	21.93 ± 2.58^a^
DHZ + TMZ	48.67 ± 4.33^b^	1.06 ± 0.17	0.015 ± 0.002	15.48 ± 1.21^b^
Glioma model				
NC	46 ± 4.712	1.78 ± 0.06	365.4 ± 12.36	8.54 ± 1.62
C6	54.20 ± 5.33	1.21 ± 0.21	139.7 ± 12.63^aaa^	23.50 ± 2.15^aaa^
C6 + TMZ	37.40 ± 3.36	1.10 ± 0.15	297.8 ± 29.66^b^	49.10 ± 7.40^bb^
C6 + DHZ + TMZ	32.33 ± 5.78	0.83 ± 0.15	465.1 ± 33.91^c^	15.80 ± 3.28^ccc^

The parameters represent the values of retention trial for 60 s in the Morris water maze apparatus. The animal movement was recorded with the camera the data were analysed using the software. All the values are expressed as mean ± SEM of six samples. The changes in the parameters of various groups are denoted by symbols. Symbols in the chemobrain model: ^a^*P* < 0.05 compared to NC, ^b^*P* < 0.05 compared to TMZ control group. Symbols in Glioma model: ^a^*P* < 0.05 compared to NC^, aa^*P* < 0.01 compared to NC, ^aaa^*P* < 0.001 compared to NC; ^b^*P* < 0.05 compared to C6 control group, ^bb^*P* < 0.01 compared to C6 control group, ^bbb^*P* < 0.001 compared to C6 control group, ^c^*P* < 0.05 compared to C6 + TMZ control group, ^cc^*P* < 0.01 compared to C6 + TMZ control group, ^ccc^*P* < 0.001 compared to C6 + TMZ control group

*NC* normal control, *C6:C6* Glioma control, *TMZ* temozolomide, *C6 + TMZ* C6 glioma + temozolomide, *DHZ + TMZ* dehydrozingerone + temozolomide

TMZ control group animals had shown a significant decrease in average speed (1.20 ± 0.04) as compared to normal control (1.74 ± 0.06). There was no significant increase in the mean speed of test group animals (Table 3).

TMZ group animals showed a significant (21.93 ± 2.58) increase in escape latency as compared to normal control (7.05 ± 0.83). DHZ + TMZ treatment had shown a significant (15.48 ± 1.21) decrease in escape latency compared to TMZ group animals (14.04 ± 1.06) (Table 3).

#### Antioxidant analysis

Due to the TMZ treatment, catalase and SOD activity was significantly decreased in both the frontal cortex (31.3 ± 0.58, 1.11 ± 0.16) and hippocampus (16.80 ± 0.97, 0.41 ± 0.02) as compared to the normal control (78.35 ± 1.76, 7.59 ± 0.72 and 58.52 ± 0.93, 9.91 ± 1.0). The treatment group, DHZ + TMZ had shown a significant increase in the activity of catalase in both the frontal cortex (32.9 ± 3.7) and hippocampus (48.32 ± 4.92) as compared to the TMZ control group. SOD levels were significantly increased in the frontal cortex (7.093 ± 0.6) as compared to the TMZ group, while a slight increase in the hippocampal region (1.95 ± 0.30) was observed (Table 4).

**Table 4:** Effect on antioxidant parameters in Chemobrain and Glioma model

Group/parameter	Catalase		SOD		LPO		GSH	
	FC	HC	FC	HC	FC	HC	FC	HC
Chemobrain model								
NC	78.35 ± 1.76	58.52 ± 0.93	7.59 ± 0.72	9.91 ± 1.0	427.1 ± 53.68	106.9 ± 31.61	119.1 ± 14.44	155 ± 9.62
TMZ	31.3 ± 0.58^aaa^	16.80 ± 0.97^aa^	1.11 ± 0.16^aaa^	0.41 ± 0.02^aaa^	1188 ± 55.34^aa^	905.7 ± 56.88^aaa^	314.8 ± 26.11^aa^	284.7 ± 18.87^a^
DHZ + TMZ	32.9 ± 3.7^bbb^	48.32 ± 4.92^bbb^	7.093 ± 0.6^bbb^	1.95 ± 0.30	717.4 ± 38.85^b^	484.7 ± 79.45^bb^	139.8 ± 18.40^b^	259 ± 40.68
Glioma model								
NC	72.44 ± 6.22	59.50 ± 1.18	11.10 ± 0.78	14.95 ± 1.07	407.3 ± 42.8	111.4 ± 22.79	118.2 ± 10.24	364.6 ± 17.62
C6	26.55 ± 4.84^aaa^	22.01 ± 4.61^a^	0.85 ± 0.30	8.19 ± 0.78	1415 ± 18^aa^	698.0 ± 48.78^aaa^	145.1 ± 2.0^aa^	327.4 ± 29.46
C6 + TMZ	32.30 ± 3.82	33.04 ± 5.36	2.23 ± 0.39	3.04 ± 1.44	3112 ± 286.6^bbb^	1149 ± 291.1^bbb^	325.4 ± 24.75^bbb^	308.6 ± 13.44
C6 + DHZ + TMZ	57.95 ± 3.56^c^	40.31 ± 9.64	1.30 ± 0.26	12.82 ± 1.11^ cc^	2021 ± 2.28.4^c^	267.0 ± 70.84^ccc^	136.0 ± 7.58^ccc^	337.3 ± 45.60

These antioxidant parameters were assessed by colorimetry. Units for various parameters are catalase (unit/mg of protein), SOD (unit/mg of protein), LPO (nM of MDA/mg of protein), GSH (µM/ mg of protein). All values are mean ± SEM of six samples. All the values are expressed as mean ± SEM. The changes in the parameters of various groups are denoted by symbols. Symbols in chemobrain model: ^a^*P* < 0.05 vs NC, ^aa^*P* < 0.01 vs NC, ^aaa^*P* < 0.001 vs NC, ^b^*P* < 0.05 vs TMZ control group ^bb^*P* < 0.01 vs TMZ control group, ^bbb^ *P* < 0.001 vs TMZ control group. Symbols in Glioma model: ^a^*P* < 0.05 vs NC^, aa^*P* < 0.01 vs NC, ^aaa^*P* < 0.001 vs NC; ^b^*P* < 0.05 vs C6 control group ^bb^*P* < 0.01 vs C6 control group, ^bbb^*P* < 0.001 vs C6 control group, ^c^*P* < 0.05 vs C6 + TMZ control group ^cc^*P* < 0.01 vs C6 + TMZ control group, ^ccc^*P* < 0.001 vs C6 + TMZ control group

*NC* normal control, *C6:C6* Glioma control, *TMZ* temozolomide, *C6 + TMZ* C6 glioma + temozolomide, *DHZ + TMZ* dehydrozingerone + temozolomide

Malondialdehyde level has significantly increased in the TMZ control group in the frontal cortex (1188 ± 55.34) and hippocampus (905.7 ± 56.88) when compared to the normal group (427.1 ± 53.68 and 106.9 ± 31.61). DHZ + TMZ treatment significantly decreased the malondialdehyde level of the frontal cortex (717.4 ± 38.85) and hippocampus (484.7 ± 79.45) as compared to the TMZ group (Table 4).

TMZ had significantly increased the GSH level in the frontal cortex (314.8 ± 26) and hippocampus (284.7 ± 18.87) in comparison with normal control (119.1 ± 14.44 and 155 ± 9.62). DHZ + TMZ treatment caused a significant decrease in the GSH level of the frontal cortex (139.8 ± 18.40), when compared to TMZ group, with slight decrease in hippocampal region (259 ± 40.68) (Table 4).

### Cognitive impairment in glioblastoma model

#### Locomotor activity

The locomotory activity of the animals was assessed by the actophotometer test in glioblastoma model. Glioma control animals had shown significant decrease in the locomotor activity (139 ± 12.63), compared to normal control animals (365 ± 12.36). DHZ + TMZ treatment showed a significant increase in locomotor activity as compared to both C6 control and C6 + TMZ control (417.3 ± 26.31) (Table 1).

#### Behavioral analysis

##### Novel object recognition test

C6 control group animals had shown a decrease in the recognition index with a novel object compared to the normal control (0.09 ± 0.09). C6 + TMZ group animals did not show any significant change in the recognition index with the novel object when compared to C6 control animals. The treatment group had shown a significant increase in the exploration time compared to the C6 + TMZ control group (0.48 ± 0.07) (Table 2).

Discrimination index is the difference in the exploration time of the novel object and the familiar object. C6 control had shown a significant decrease in the discrimination index (−0.51 ± 0.3). as compared to normal control animals (3.55 ± 0.57). C6 + TMZ group (− 0.69 ± 0.29) did not show any significant decrease in discrimination of novel object from the familiar object when compared to C6 group. The treatment group, DHZ + TMZ (3.23 ± 0.60) had shown a significant increase in the discrimination of novel object from the familiar object as compared to the C6 control and C6 + TMZ control group (Table 2).

#### Morris water maze test

C6 + temozlomoide did not show any significant difference in total zones entries as compared to the normal control group. None of the treatment groups showed a significant difference in the total zones entries.

There was no significant increase in the mean speed of C6 + TMZ control group animals as compared to the C6 control group animals. None of the treatment groups showed a significant difference in average speed.

C6 + TMZ control had shown a significant (297.8 ± 29.66) decrease in path efficiency as compared to C6 control (139.7 ± 12.63). DHZ + TMZ treatment had shown a significant (465.1 ± 33.91) increase in the path efficiency when compared to C6 + TMZ control.

C6 control had shown a significant increase in escape latency (23.50 ± 2.15) as compared to normal control (8.54 ± 1.62). C6 + TMZ control group animals had shown a significant (49.10 ± 7.401) increase in escape latency, when compared to the C6 control while it was reversed in the C6 + DHZ + TMZ group animals (15.80 ± 3.28) (Table 3).

#### Estimation of antioxidant parameters in the glioblastoma model

In the frontal cortex, C6 control group animals showed a significant decrease in catalase activity (26.55 ± 4.84) as compared to normal control (72.44 ± 6.22). GSH in C6 + TMZ group (32.30 ± 3.82) did not show any significant change as compared to C6 control group. DHZ + TMZ treatment had shown a significant (*P* < 0.05) increase in catalase activity (57.95 ± 3.56) in the frontal cortex as compared to the C6 + TMZ control. In the hippocampus, a similar trend was observed like the frontal cortex catalase level. A significant (*P* < 0.05) increase in catalase levels was observed in the C6 control group (22.01 ± 4.61) as compared to the normal control group (59.50 ± 1.18). No significant change was observed in the catalase levels between C6 + TMZ group (33.04 ± 5.36) and C6 + DHZ + TMZ group (40.31 ± 9.64).

The SOD activity of the frontal cortex and hippocampus had significantly decreased (0.85 ± 0.30, 8.19 ± 0.78) in C6 control group animals when compared to normal control (11.10 ± 0.78, 14.95 ± 1.07). TMZ did not show any significant decrease in SOD levels of the frontal cortex and hippocampus as compared to C6 control group animals. DHZ did not improve the SOD levels as compared to C6 + TMZ group in the frontal cortex (1.30 ± 0.26) while a significant increase was observed in the hippocampus(12.82 ± 1.11) as compared to C6 + TMZ group (2.23 ± 0.39, 3.04 ± 1.44).

In the frontal cortex and hippocampus, the C6 control group had shown a significant increase in LPO level (1415 ± 18, 698.0 ± 48.78) as compared to the normal control (407.3 ± 42.8, 111.4 ± 22.79). C6 + TMZ control group animals had shown a significant increase in the level of LPO as compared to C6 control in the frontal cortex and hippocampus. DHZ + TMZ treatment significantly decreased the LPO level (3112 ± 286.6, 1149 ± 291.1) in the frontal cortex and hippocampus (2021 ± 2.28, 267.0 ± 70.84) as compared to C6 + TMZ control.

In the frontal cortex, GSH levels of the C6 control group (145.1 ± 2.0) showed a significant (*P* < 0.01) increased as compared to the normal control group (118.2 ± 10.24). GSH of C6 + TMZ control (325.4 ± 24.75) showed a significant increase in GSH levels as compared to the C6 control group, which was reversed by the treatment group, C6 + DHZ + TMZ (136.0 ± 7.58). In the hippocampus, no statistically significant change was observed among the groups (Table 4).

#### Tumor parameters

Tumor volume and tumor weight were evaluated after sacrificing the animals. A significant reduction in tumor volume and tumor weight was observed in the TMZ group compared to the C6 control group. Co-treatment with DHZ with TMZ significantly reduced the tumor volume and tumor weight compared C6 group and TMZ group (Table 5).

**Table 5:** Effect on tumor volume

Groups	Tumor volume (mm^3^)	Tumor weight (g)
C6	102.95 ± 7.59	0.159 ± 0.02
C6 + TMZ	58.90 ± 6.30^bb^	0.085 ± 0.003^bb^
C6 + DHZ + TMZ	30. 79 ± 6.92^bbb,c^	0.04 ± 0.002^bbb,c^

A significant decrease in tumor volume and tumor weight was observed by TMZ and TMZ + DHZ. The changes are represented as: ^bb^*P* < 0.01 compared to C6 control, ^bbb^P < 0.001 compared to C6 control, and ^c^*P* < 0.05 compared to C6 + TMZ control. All values are Mean ± SEM of six animals. The symbols are: C6:C6 Glioma control; C6 + TMZ: C6 glioma + temozolomide; DHZ + TMZ: dehydrozingerone + temozolomide

#### Histology of cortex

**Fig. 3:**
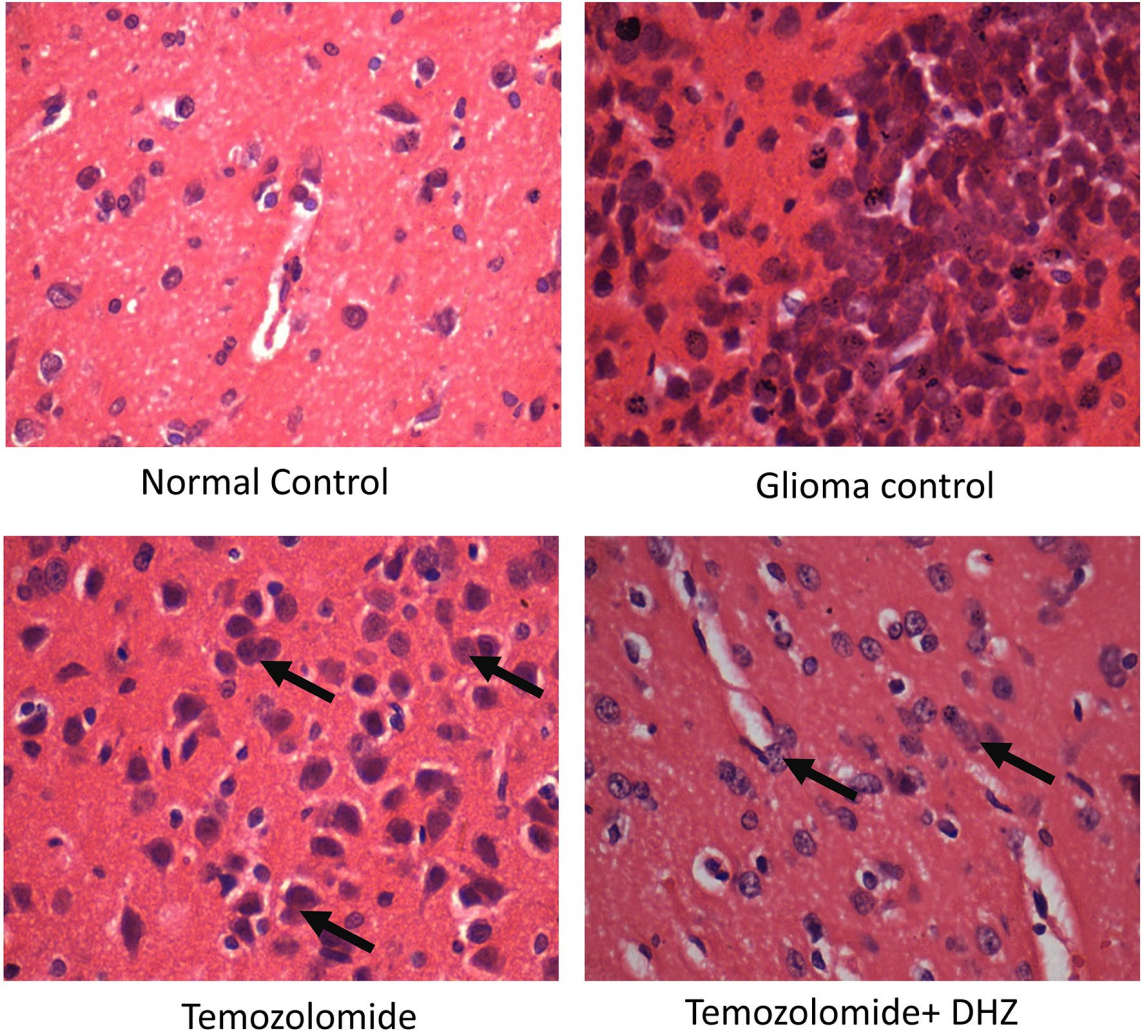
Histology of cortex in the glioma model. Photomicrographs showing H&E images of the cerebral cortex (10×); the cerebral cortex of glioma rats showed increased astrocytes with bi-nucleus and clusters of large neurons compared to the cerebral cortex of the control. Low-grade glioma was observed in the cerebral cortex of both the treated groups: TMZ and TMZ + DHZ. Fibrosis, necrosis, and calcification were not observed in any of the treated groups. The black arrow represents proliferating astrocytes

Histology of the cerebral cortex of glioma rats showed increased astrocytes with bi-nucleus and clusters of large neurons compared to the cerebral cortex of the control. Treatment with TMZ showed a low-grade glioma in the cerebral cortex in comparison to C6 glioma control. The addition of DHZ with TMZ showed a decrease in glioma cells. Fibrosis, necrosis, and calcification were not observed in any of the treated groups (Fig. 3).

#### Histology of hippocampus

**Fig. 4:**
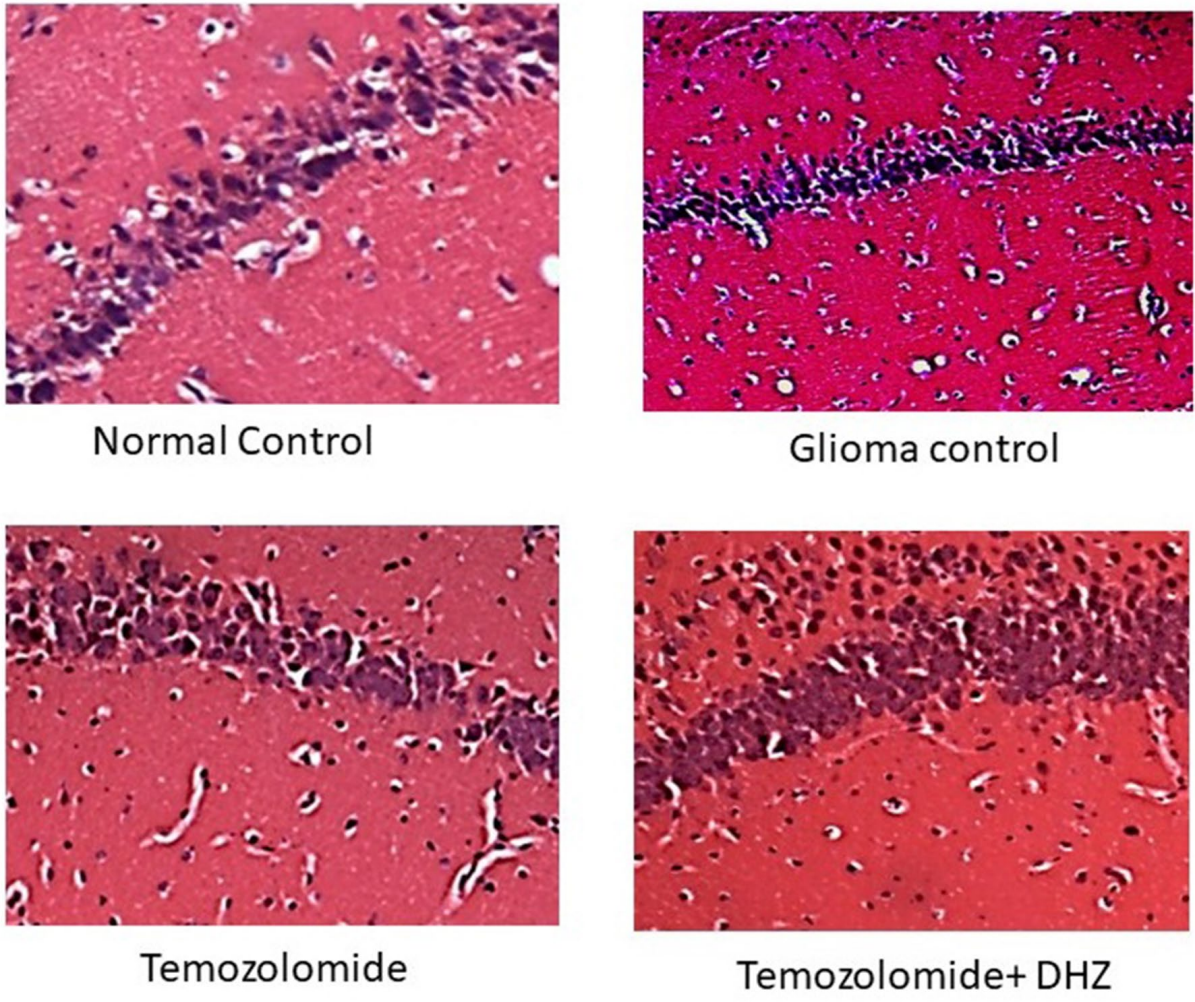
Histology of hippocampus in glioma model. Photomicrographs showing the H&E section of rat hippocampus CA3 region (10×); the normal architecture of CA3 region with large pyramidal cells is well maintained in all the treated groups when compared to control. No apoptosis or clumping of neuronal nerve fibers was observed in any of the treated groups

CA3 region of the hippocampus did not show any alteration in C6 glioma rats. The normal architecture was found in the CA3 region with large pyramidal cells. This was well maintained in all the treated groups when compared to control. The results indicated the localisation of tumors to the cortex only. No apoptosis or clumping of neuronal nerve fibers with the TMZ or TMZ + DHZ treatment were observed (Fig. 4).

## Discussion

Chemotherapy mediated neurotoxicity is manifested by impaired attention, executive functions, memory, etc., even after cessation of therapy, consequently compromising the quality of life. This is termed as ‘chemo-fog’ or ‘chemo-brain’ (Mrugala and Chamberlain 2008; Dietrich et al. 2015b) However, not much evidence is available for TMZ. Thus, the present study was designed to explore the chemobrain condition induced by TMZ in Male Wistar rats in the normal and, in the animals with glioblastoma. Flavonoid namely, DHZ was selected based on its earlier reports on chemoprevention, antioxidant, and anti-inflammatory properties (Motohashi et al. 1997).

TMZ-induced cognitive impairment was developed in male Wistar rats by administration of TMZ 18 mg/kg i.v*.* once in 5 days over 32 days. Based on previous reports, DHZ was given at a dose of 100 mg/kg p.o. daily (Wick et al. 2009; Anusha et al. 2016). The cognitive function was assessed for episodic and spatial memory by NORT (Cheruku et al. 2019) and Morris water maze test, respectively (Nampoothiri et al. 2017). To assess the toxic effect of TMZ on locomotor activity, an actophotometer was used.

In the chemobrain model, TMZ treated animals showed significantly less exploration time to the novel object than the familiar object when compared to the normal control animals. This was evident by a significantly lesser recognition index (RI) and discrimination index (DI) in comparison to normal control animals. Similar reports are available for doxorubicin with a difference in the intertrial interval (ITI). The ITI in doxorubicin was found to be 4 h, while for TMZ, ITI was kept 12 h (Cheruku et al. 2018, 2019). Animals from the DHZ treatment group spent significantly more time exploring the novel object compared to the TMZ alone treatment group, which was evident by a significant improvement in RI and DI, compared to the TMZ control group.

Morris Water Maze test was performed to assess the spatial memory of the animals. Escape latency is one of the important parameters of the Morris water maze test and it was performed to understand the loss of memory in the animals (Cheruku et al. 2018; Mueller et al. 2010). The other important parameter which reflects the memory component and locomotion are total zone entries and path efficiency. In the chemobrain model, the TMZ control animals showed a significant decrease in total zones entries, average speed, and path efficiency compared to the normal control group reflecting cognitive impairment by TMZ affecting spatial memory. The DHZ treatment group showed a significant increase in the total number of zones entries and decreased escape latency indicating the ability of DHZ to protect against TMZ induced cognitive impairment. The rest of the parameters were not significantly altered, compared to TMZ control.

The locomotor activity of the animals was evaluated to assess the effect of TMZ on motor function. None of the treatment groups showed any significant difference in the locomotor activity of the rats. This indicated the change in RI and DI is mainly attributed due to cognitive impairment and not to the motor impairment.

Glioma was induced by injecting 50,000 C6 glial cells intracerebrally into the brain of the animals (Ozeki et al. 2012) and observed over 7 days for the recovery of animals from surgery and the development of the tumor thereafter dosing and treatment schedule was followed similar to chemobrain model. The locomotor activity of the C6 control group was significantly decreased when compared to the normal control group. C6 + TMZ control group was showing a significant increase in the locomotor activity as compared to the C6 control. Due to the treatment with TMZ and DHZ, there was a significant increase in the locomotor activity of the animals as compared to the C6 control group. This reflected that the tumor burden has decreased locomotor activity and the TMZ and drug treatment reversed the effect of cancer on locomotion as observed by actophotometer.

An episodic and special memory was effectively restored by DHZ treatment. The results on cognitive impairment by TMZ were supported by the reports from Nokia et al. (2012). TMZ is readily permeable through BBB, as a result, it produces direct toxicity to the normal cells of the brain leading to impaired hippocampal neurogenesis, and disruption in the associative learning. In the adult brain, the hippocampus is responsible for the formation and retention of episodic and spatial memory (Burgess et al. 2002). Hippocampal neurogenesis in the adult brain occurs by the process of generating new granule cell neurons. This process regulates the maintenance of brain plasticity, learning, and memory. TMZ treatment for glioblastoma is implicated in the impairment of hippocampal neurogenesis showing a disruption in theta activity of the brain which determines the decline in learning and memory and disturbance in communication between brain centers, i.e., inter-regional communication, confirming the impairment in cognitive ability (Nokia et al. 2012; Dietrich et al. 2015a). Further mechanistic evaluation for neuroprotection was performed by antioxidant analysis.

Tumor cells are more dependent on the GSH level in glioma. The mechanism of resistance to TMZ is considered to be by increased GSH levels (Rocha et al. 2016). It is also found that depletion in GSH levels worked out in favor of decreasing the resistance to TMZ. In the present study, similar results were found regarding GSH level, it was elevated in Glioblastoma and further got elevated by TMZ treatment. A significant decrease was observed by the DHZ in both models, normal as well as glioblastoma models. Other Antioxidant parameters followed the reverse trend in both models by the treatment.

In the first model, due to the TMZ treatment, the catalase and SOD levels were significantly decreased in the frontal cortex and hippocampus. This effect was significantly reversed by the treatment group (DHZ + TMZ). Temozolomide is known to increase the intracellular ROS levels leading to autophagy and apoptosis in cancer cells. However, in normal conditions, a persistent increase in the ROS levels due to repeated administration of TMZ may have reduced the catalase and SOD activity in the hippocampus and frontal cortex (Lo Dico et al. 2019). MDA levels indicate lipid peroxidation due to the generation of free radicals (Rai et al. 2019). The MDA levels in the present study showed a significant increase in the hippocampus and frontal cortex due to temozolomide treatment whereas the treatment group showed a significant decrease in the MDA levels in the hippocampus and frontal cortex.

In the glioblastoma model, the catalase activity of the frontal cortex and hippocampus was significantly decreased in the rats of C6 control group as compared to the normal control group. DHZ + TMZ treatment significantly increased the catalase level in the frontal cortex. Superoxide dismutase activity was significantly decreased in the C6 control group when compared to the normal control group. The treatment group showed a significant increase in SOD activity. LPO levels significantly increased in C6 + TMZ control, compared to C6 control. The treatment (DHZ + TMZ) showed a significant decrease in the MDA levels of the frontal cortex and hippocampus, as compared to the C6 + TMZ control group.

The antioxidant estimation of various groups supported the hypothesis. An increase in the levels of antioxidants like SOD and catalase, both in the frontal cortex and hippocampus in treatment groups, showed that DHZ is actively reversing the cell damage caused by both cancer and TMZ. A significant increase in MDA levels showed that the cell damage caused due to cancer development and TMZ treatment was successfully reversed with DHZ treatment, proving its potent anti-oxidant activity.

Overall, the study focused on reversing the cognitive impairment caused by TMZ in normal and in the presence of glioma, by treating with a half analog of well-known antioxidant curcumin i.e. DHZ. The major mechanism behind the antioxidant property of DHZ is the presence of the phenolic group in its structure. The phenolic group is attached to the para position in the molecule. Phenols are already proven to have good antioxidant properties as they have the ability to form phenoxy radicals by reacting with the free radicals in the brain, generated by the underlying disease condition or TMZ treatment. DHZ also possesses a methoxy group in ortho position to phenolic group which increases the free radical scavenging ability by the inductive effect. Previous studies have quoted the importance of the presence of electron-donating groups such as the methoxy group, in increasing the antioxidant property of phenol. In addition to this, the presence of a double bond in conjugation with phenyl ring increases the electron delocalization, hence enhances the antioxidant effect of DHZ (Rao 1994; Graf 1992).

In conclusion, the present study showed that DHZ protects against the cognitive impairment induced by treatment with TMZ in normal and C6- induced glioma rats. Further studies can be done to explore the mechanism of DHZ in preventing resistance development agaisnt TMZ in glioma management and to explore the clinical effectiveness of this combination in the management of glioblastoma.
